# Intestinal Stem Cells From Patients With Inflammatory Bowel Disease Retain an Epigenetic Memory of Inflammation

**DOI:** 10.1016/j.jcmgh.2026.101774

**Published:** 2026-03-27

**Authors:** Feda H. Hamdan, Mona Farhadipour, Isaiah Perez, Kimberlee Kossick, Hannah Smith, Adam Edwinson, Jose M. de Hoyos-Vega, Michelle Gonzalez, David Chiang, Emily Klatt, Kristy Rumer, Mauricio Perez Pachon, Mary Sagstetter, Jessica Friton, Erin Kammer, Alana English, Lucas C.S. Chini, Jarl F. Carnahan, Noah A. Baca, Jessie Hohenstein, Rohini Mopuri, Aditya Bhagwate, Laura E. Raffals, Rondell Graham, Zhifu Sun, Madhusudan Grover, Alexander Revzin, Michael Kattah, William A. Faubion, Brooke R. Druliner

**Affiliations:** 1 Division of Gastroenterology and Hepatology, Mayo Clinic, Scottsdale, Arizona; 2 Division of Gastroenterology and Hepatology, Mayo Clinic, Rochester, Minnesota; 3 Department of Physiology and Biomedical Engineering, Mayo Clinic, Rochester, Minnesota; 4 Department of Colon and Rectal Surgery, Mayo Clinic, Rochester, Minnesota; 5 Division of Quantitative Health Sciences, Mayo Clinic, Rochester, Minnesota; 6 Division of Anatomic Pathology, Mayo Clinic, Rochester, Minnesota

**Keywords:** Chromatin, Epigenetics, Inflammatory Bowel Disease, Intestinal Stem Cells, Organoids

## Abstract

**Background & Aims:**

Intestinal epithelial damage and impaired repair are hallmarks of ulcerative colitis (UC), even after inflammation resolves. Intestinal stem cells (ISCs) can retain stable epigenetic changes after inflammation, highlighting the potential for long-lived epithelial memory in the gut. Inflammatory injury in barrier tissues induces epigenetic memory in epithelial stem cells, and the tendency of UC to relapse at previously inflamed sites led us to hypothesize that ISCs from patients with inflammatory bowel disease acquire lasting memory of prior inflammation.

**Methods:**

To test this, we derived colonic organoids from inflamed (I) and noninflamed regions of the same patients with UC and propagated in long-term culture.

**Results:**

Chromatin profiling revealed 2252 accessible regions unique to I organoids, associated with stress response, repair, and inflammatory genes. Although these regions remained accessible, ∼95% of associated genes were not upregulated in I organoids, indicating a primed state. Upon inflammatory or injury re-challenge, I organoids exhibited heightened transcriptional responses and accelerated wound closure, despite reduced clonogenicity and impaired barrier function, indicating a retained inflammatory memory program.

**Conclusions:**

Our findings demonstrate that human ISCs retain a chromatin-based memory of inflammation that persists in the absence of immune cues and shapes future responses to injury. Although this may support epithelial adaptation to secondary insults, it may predispose tissue to relapse in patients with UC.

## Graphical abstract

**Figure:**
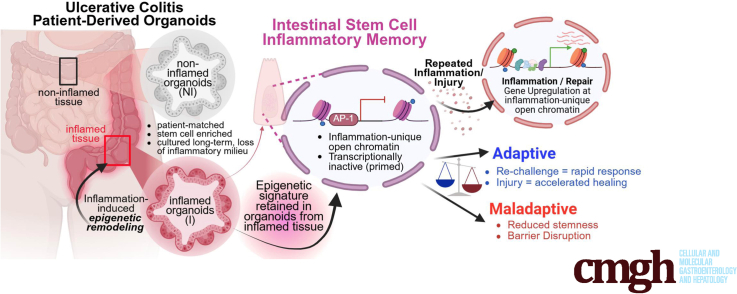

**Summary**

Human intestinal stem cells from ulcerative colitis patients retain a chromatin memory of inflammation. This primed epigenetic state contributes to altered stem cell function at baseline and a rapid response to injury, providing a mechanism for persistent epithelial dysfunction despite the resolution of inflammation.

**What You Need to Know**

**Background**

Intestinal stem cells regenerate the epithelium in homeostasis and repair after injury, but it is unclear how prior inflammation shapes their long-term behavior.

**Impact**

We show that human intestinal stem cells retain a chromatin memory of prior inflammation that primes them for accelerated responses upon re-injury and contributing to stem cell dysfunction, providing a mechanism for persistent altered mucosal healing in ulcerative colitis.

**Future Directions**

Defining mechanistic regulators that establish and maintain inflammatory memory in intestinal stem cells may explain why ulcerative colitis recurs, even after apparent healing. This insight could guide therapeutic strategies with the goal of improving epithelial repair and reducing disease recurrence.

Inflammatory bowel disease (IBD) comprises chronic, relapsing disorders of the gastrointestinal tract, including ulcerative colitis (UC), which is a relapsing and remitting disease with little insight into the mechanisms governing recurrent cycles of inflammation and impaired mucosal healing.^1^, ^2^, ^3^ Despite therapeutic advances, only 30% to 35% of patients experience sustained clinical remission, and most patients suffer >3 relapses per year.^4^, ^5^, ^6^, ^7^ UC is unique in that 15% to 20% of patients experience limited disease with sharp demarcation between continuous inflammation and histologically normal mucosa.^8^ Although the pathophysiologic explanation of such demarcated regions of inflammation adjacent to histologically normal mucosa is unknown, this feature provides an opportunity to study uninflamed and inflamed tissue in the same patient. Current therapies focus exclusively on suppressing immune activity, with the goal of reducing inflammation to allow the intestinal epithelium to repair. Incomplete mucosal healing persists in many patients despite remission, highlighting the need to define how chronic inflammation affects epithelial repair.^9^^,^^10^

The intestinal epithelium is one of the most rapidly renewing tissues in the body, undergoing complete turnover every 3 to 5 days. This regenerative capacity is fueled by intestinal stem cells (ISCs), located at the base of the crypts, which maintain epithelial homeostasis and mount rapid repair responses following injury. In response to inflammation, infection, or physical damage, ISCs activate stress-response programs that promote proliferation and coordinate epithelial regeneration.^11^, ^12^, ^13^ Although the intestinal epithelium often appears histologically normal following resolution of inflammation, IBD is marked by a relapsing-remitting disease course, suggesting that full molecular recovery may not always accompany morphological healing. This raises the possibility that inflammation leaves behind a molecular memory of that experience in ISCs that influences their future behavior.

Emerging studies in IBD have shown that the intestinal epithelium can retain stable epigenetic modifications after inflammatory exposure. In Crohn’s disease, patient-derived organoids display persistent hypomethylation at MHC class I loci, linked to altered epithelial-immune interactions.^14^ In a murine model of gastrointestinal graft-vs-host disease, intestinal stem cells exhibit durable chromatin and DNA methylation changes that impair regenerative capacity.^15^ These findings support the broader concept that epithelial memory may contribute to disease pathogenesis in the gut.

Although memory in immune cells has been recognized for decades as central to host defense,^16^, ^17^, ^18^, ^19^, ^20^ recent studies in murine skin have established that nonimmune cells such as epithelial stem cells can also retain stable epigenetic changes induced by prior inflammation.^21^ This form of long-lasting epigenetic reprogramming is called inflammatory memory, where inflammatory insults can induce lasting changes in chromatin accessibility, leaving stem cells in a primed transcriptional state. This memory is mediated by transcription factors such as activator protein 1 (AP-1; FOS/JUN) and signal transducer and activator of transcription 3 (STAT3), which preserve accessibility at specific enhancer regions and enable rapid reactivation of target genes during subsequent injury.^22^ Inflammatory memory has also been observed in other tissues, including the pancreas and developing intestine following early-life infection, suggesting it may represent a conserved epithelial response to injury.^23^, ^24^, ^25^ Understanding how prior inflammation alters ISC function may help explain why epithelial repair remains incomplete in many patients with UC, even during periods of clinical remission, and may offer insight into mechanisms driving relapse. The contribution of inflammatory memory to ISCs, and the consequences for the intestinal epithelium in chronic inflammatory disease such as IBD is the central aim of our study.

To investigate how inflammatory exposures affect ISC molecular and functional properties, we used intestinal organoids, 3-dimensional cultures that preserve epithelial diversity and support long-term ISC expansion.^26^, ^27^, ^28^, ^29^ Organoids provide a human epithelial model system to study IBD, independent of immune signals.^30^, ^31^, ^32^, ^33^, ^34^ Prior studies have shown that organoids can preserve transcriptional programs reflective of their tissue of origin, including disease-associated gene expression patterns.^35^ Although emerging evidence from cancer and developmental models suggests that organoid systems can retain epigenetic features, this has not been systematically examined in the context of IBD.^35^, ^36^, ^37^ In the present study, we generated patient-matched organoids from inflamed (I) and uninflamed (noninflamed [NI]) colonic tissue in individuals with UC, enabling direct, intra-patient comparisons of epithelial state. By integrating chromatin and transcriptional profiling, we identify a conserved program of inflammatory memory in human ISCs that persists after inflammation resolves and alters subsequent regenerative responses of the ISC.

## Results

### Inflammation Alters the Phenotype of ISCs in Human Organoids

**Figure 1:**
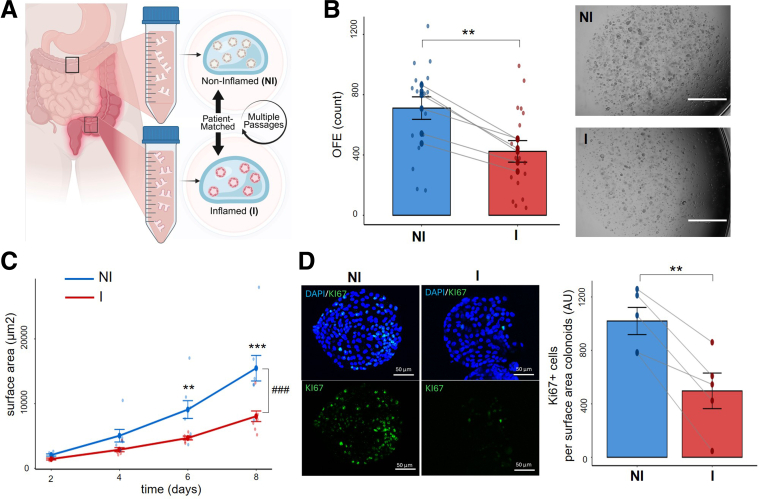
**Patient-derived organoids from inflamed tissue show altered stem cell function compared to matched organoids from uninflamed sites.** (*A*) Schematic of paired sampling strategy showing I and NI regions of the colon from the same patient with UC. Organoids are cultured under identical conditions and are passaged several times to remove inflammatory environment before any molecular profiling. (*B*) OFE is reduced in I compared with NI organoids. Data represent the number of organoids generated from 50,000 single cells dissociated from paired organoids lines, representing predominantly undifferentiated crypt base cells. (*C*) Time-dependent changes in the surface area indicate that I organoids grow slower than NI organoids over 8 days of culture (n = 6). (*D*) The number of proliferative cells (Ki67^+^) is reduced in I compared with NI organoids, normalized to organoid surface area, on day 8 prior to passaging. Representative immunofluorescence images show DAPI (*blue*, nuclei) and Ki67 (*green*, proliferating cells). For all panels, n represents the number of patient lines for which matched NI and I cultures were assessed. Data represent mean ± SEM. For (*B* and *C*), data are pooled from 3 consecutive passages. ∗∗*P* < .01; ∗∗∗*P* < .001 vs NI; ^###^*P* < .001 (time × condition). LMM with fixed factors: condition, passage, and time; random factor: patient.

To examine how prior inflammatory exposure influences ISC behavior, we established paired human colonic organoids from I and NI regions of the same patients with UC, N = 6 (Figure 1*A*). We refer to organoids from I tissue as “inflamed” because they were isolated from sites of inflammation, but culture through sufficient passage outside the inflammatory milieu represents a prior inflamed state, enabling us to study of the lasting epithelial-intrinsic effects of past inflammation. Although previous studies have successfully derived organoids from patients with IBD, few have generated organoids from actively inflamed tissue, and to our knowledge no published study has performed matched comparisons of I and NI regions within the same individual.^35^, ^36^, ^37^ This model enables direct intra-patient comparison while controlling for genetic and environmental variation, allowing us to assess persistent, epithelial-intrinsic changes in stem cell function following inflammation. Once established in vitro, all organoids were maintained under identical, stem cell-preserving conditions and passaged at least 10 times to eliminate residual immune and stromal influences, ensuring that any lasting molecular or functional differences reflected stable epithelial changes rather than transient inflammatory effects. To confirm at the molecular level that the tissues used to establish organoids reflected active inflammation, we compared gene expression between paired I and NI primary tissues available from 3 of the 6 patients with UC included in this study (n = 3 patient tissue pairs, NI and I). I UC tissues demonstrated significantly higher expression of inflammatory markers compared with their matched NI counterparts (Supplementary Figure 1*A*). This was quantified using an inflammatory composite score comprised of canonical proinflammatory genes, which together reflect active mucosal inflammatory signaling at the time of organoid generation.

To evaluate the growth and regenerative capacity of ISCs from inflamed and noninflamed regions, we measured organoid-forming efficiency (OFE), surface area kinetics, and proliferation of the organoids. OFE serves as a functional readout of stem cell fitness and regenerative potential through the measure of the fraction of single cells that successfully establish and sustain organoid growth in culture, whereas surface area provides a morphological assessment of stem cell function over time in culture.^38^ We performed these assays in all patients (n = 6), across 3 passages. We found that I organoids exhibited a significantly reduced OFE compared with NI organoids (Figure 1*B* and Supplementary Figure 1*B* and *C*). Across 8 days of culture, I organoids consistently showed reduced size, with surface area significantly decreased (Figure 1*C*, Supplementary Figure 1*D* and *E*). To assess proliferative capacity, whole-mount immunofluorescence analysis revealed a significant reduction in KI67 cells in I compared with NI organoids (Figure 1*D*).

**Figure 2:**
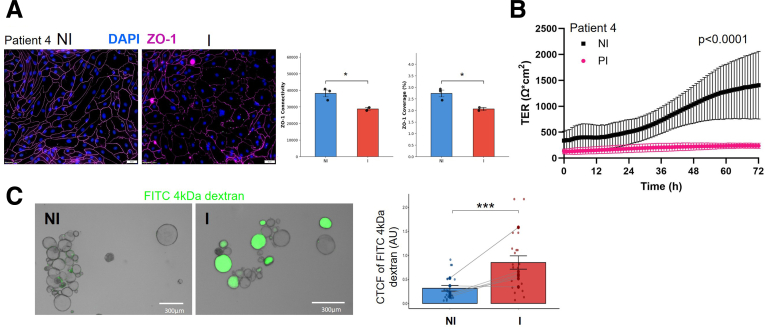
**Organoids from inflamed tissue have impaired barrier integrity.** (*A*) Immunofluorescence staining of ZO-1 reveals disrupted tight junctions in I monolayer compared with NI monolayer. Representative images on the left show ZO-1 (*pink*) and nuclei (DAPI, *blue*). Quantification of ZO-1 connectivity on right. ∗*P* = .03. (*B*) TER is significantly decreased in I epithelial monolayers (*red*) compared with NI (*blue*) over 3 days of culture, performed in 3 technical replicates. (*C*) FITC-dextran (4 kDa) permeability is increased in I organoids compared with NI (n = 6). Representative images show dextran entry into the organoid lumen with corresponding quantification of CTCF. For all panels, n represents the number of patient lines for which matched NI and I cultures were assessed. Data represent mean ± SEM. For (*C*), data are pooled from 3 consecutive passages. ∗∗*P* < .01; ∗∗∗*P* < .001 vs NI. LMM with fixed factors: condition, passage; random factor: patient.

We and others have reported disruptions in barrier proteins in IBD following inflammation,^39^, ^40^, ^41^ and indeed, the I organoids showed that the tight junction protein Zonula Occludens-1 (ZO-1) was disrupted compared with NI organoids (Figure 2*A* and Supplementary Figure 2*A*). This disruption in ZO-1 was confirmed as functional by measuring lower transepithelial resistance (TER) in the prior inflamed (PI) organoids compared with NI (Figure 2*B*), indicating barrier disruption. Molecular permeability was assessed in 3D organoids using fluorescein isothiocyanate (FITC)-dextran (4 kDa) as a tracer (Figure 2*C*, Supplementary Figure 2*A* and *B*). I organoids exhibited a significant increase in fluorescence intensity relative to NI organoids, confirming disrupted junctional integrity. These results indicate that I organoids exhibit compromised barrier function at both ionic and molecular levels. Together, these findings demonstrate that ISCs retain functional hallmarks of prior inflammation, even after prolonged culture in the absence of inflammatory cues.

### ISCs Retain an Epigenetic Signature of Prior Inflammation

**Figure 3:**
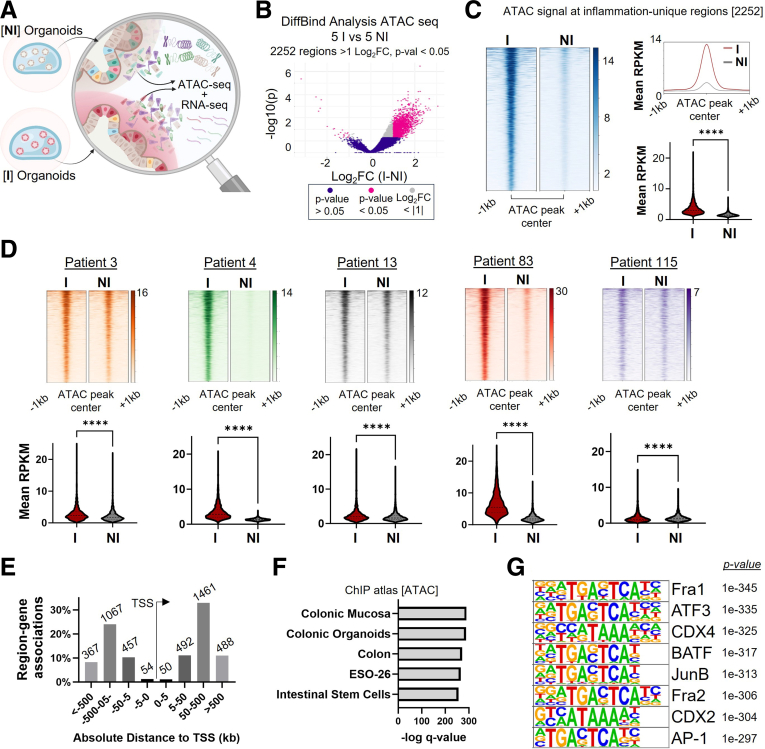
**Chromatin accessibility is altered in ISCs from I organoids.** (*A*) Schematic of sequencing performed in NI and I organoids; these are the identical organoids used throughout. (*B*) Volcano plot showing 2252 I-unique accessible regions (*pink*) consistently across multiple patients following analysis of differential chromatin accessibility between NI and I organoids. (*C*) Mean ATAC-seq signal at I-unique peaks demonstrates significantly sustained accessibility in I compared with NI. (*D*) Heatmaps of ATAC-seq signal centered on I-unique peaks. Violin plots of mean RPKM values of ATAC peaks indicate statistically significant differences (∗∗∗∗*P* < .0001). (*E*) Genomic annotation of open chromatin regions. (*F*) ChIP Atlas annotation of I-unique ATAC regions showing significant enrichment in intestinal stem cell and colonic organoid datasets. (*G*) Transcription factor motif enrichment analysis highlights AP-1 family members (JUNB, ATF3) and lineage-specific factors (CDX2) in I-unique regions.

The sustained epithelial dysfunction observed in I organoids, even after removal from the inflammatory environment, prompted us to hypothesize that prior inflammation may reprogram ISC chromatin, similar to what has been reported in epidermal stem cells following inflammation.^21^^,^^22^ To investigate this, we performed assay for transposase-accessible chromatin using sequencing (assay for transposase-accessible chromatin using sequencing [ATAC-seq]) to globally profile chromatin accessibility in ISCs preserved in I and NI organoids (Figure 3*A*). This approach provides an epigenetically unbiased view of the genome-wide accessible chromatin landscape, enabling the identification of regulatory regions altered by prior inflammation without requiring prior knowledge of specific epigenetic marks or regulatory elements.

Analysis of differential chromatin accessibility revealed 2252 chromatin regions (inflammation-unique regions) that were more open in I relative to NI organoids, and that were consistent across all patients (Figure 3*B*). Correlation analysis showed strong concordance between duplicates and a higher agreement between patients over inflammation status (Supplementary Figure 3*A*), whereas principal component analysis demonstrated a clear separation between I and NI organoids (Supplementary Figure 3*B*). When examining Inflammation-unique regions, I organoids demonstrated significantly higher chromatin accessibility compared with matched NI organoids, both when aggregated as consensus peaks across patients (Figure 3*C*) and when analyzed individually by patient (Figure 3*D* and Supplementary Figure 3*D*). Enrichment of I-specific ATAC peaks was observed in all 5 independent patient pairs, with consistent directionality despite variability in effect, highlighting the reproducibility of this chromatin signature across individuals.

The inflammation-unique chromatin regions were enriched primarily at enhancer regions (Figure 3*E*), which is reflected in the representative genome tracks showing sustained increased accessibility at enhancer regions at the C-X-C motif chemokine ligand 2 (CXCL2) and fibroblast growth factor 2 (FGF2) loci in I organoids (Supplementary Figure 3*D*). We also observed similar chromatin accessibility in NI-I organoids pairs at epithelial cell adhesion molecule (EPCAM), a gene essential for epithelial identity and integrity, illustrating that key regulatory regions required for core epithelial function remain accessible despite prior inflammatory exposure (Supplementary Figure 4*A*). Overlapping our set of inflammation-unique regions with the chromatin immunoprecipitation (ChIP) atlas database^42^ specific to publicly available ATAC-seq data revealed the specificity of our system, including ISCs and colonic organoids (Figure 3*F*). To identify regulatory factors that may contribute to or result from persistent chromatin accessibility in PI ISCs, we performed motif enrichment analysis,^43^ motif footprinting,^44^ and co-localization enrichment^45^^,^^46^ at the I-unique open chromatin regions, which revealed strong enrichment for AP-1 factors (JUNB, ATF3), along with lineage-specific binding motifs (CDX2), which was consistent using multiple different programs (Figure 3*G*, Supplementary Figure 4*B* and *C*). The motifs enriched in NI organoids suggest that noninflamed ISCs maintain transcriptional programs associated with homeostasis, epithelial barrier maintenance, and metabolic stability, with reduced accessibility in I organoids perhaps reflecting a disruption of normal epithelial identity and function. These results demonstrate that prior inflammation leaves a lasting chromatin imprint in ISCs, characterized by sustained accessibility at enhancer regions and enrichment of AP-1 and intestinal lineage transcription factor motifs, supporting the existence of an epigenetic memory of intestinal inflammation.

### Inflammation-Associated Open Chromatin Reflects a Transcriptionally Primed State

**Figure 4:**
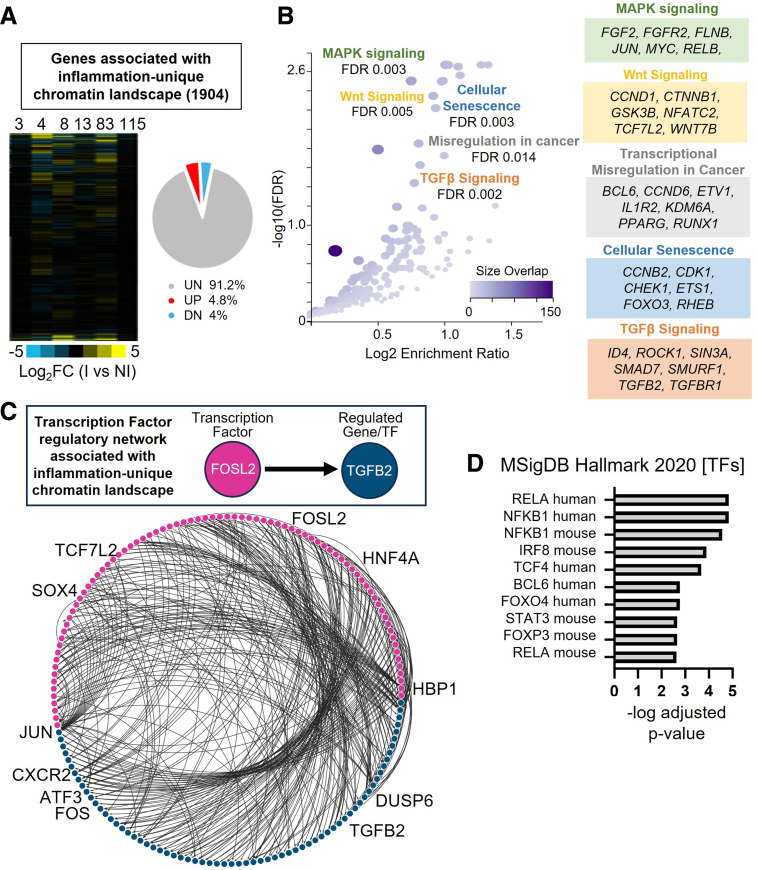
**Genes linked to inflammation-unique chromatin regions reflect modest transcription activation.** (*A*) Heatmap of Z-score FPKM expression values for 1904 genes associated with Inflammation-unique chromatin regions across 6 patients (*left*). Pie chart illustrating the percentage of genes that were unchanged (UN), upregulated (UP), or downregulated (DN) when comparing I with NI at the 1904 genes. (*B*) Pathway enrichment of Inflammation-associated genes using KEGG. (*C*) Transcription factor (TF) regulatory network reveals connectivity among TFs and their associated genes. Top box shows an example of iRegulon approach to determining regulation of TF with an associated gene. A fully annotated network plot is located in Supplementary Figure 5*A*. (*D*) Comparison of I-unique regions specifically with transcription factor target gene enrichment from MSigDB.

To assess whether the persistent open chromatin regions identified in I organoids were linked to transcriptional changes, we performed bulk RNA sequencing (RNA-seq) on the same I and NI organoids. To integrate this data with our ATAC-seq results, we mapped the 2252 PI-unique open chromatin regions to their associated genes,^47^ which resulted in 1904 protein coding genes associated with the inflammation-unique chromatin landscape. We then examined transcriptional differences between I and NI organoids across all patients. Despite marked differences in chromatin accessibility, few of the genes associated with accessible chromatin were differentially expressed between I and NI organoids (Figure 4*A*). Only 4.8% of genes were significantly upregulated by differential expression analysis in all patients (fold change ≥1; *P*-adjusted < .05). This lack of transcriptional activation despite increased chromatin accessibility suggests that the regions are in an inactive, but primed regulatory state. Pathway enrichment analysis of genes linked to inflammation-unique chromatin regions revealed significant overrepresentation of biological programs associated with epithelial stress responses, regeneration, and inflammation, including mitogen-activated protein kinase (MAPK), WNT, transforming growth factor β (TGFβ) signaling, and cellular senescence (Figure 4*B*).

To further investigate the transcriptional regulators driving these programs, we performed a transcription factor regulatory network analysis, which maps transcription factors to their putative target genes based on motif enrichment and cis-regulatory sequence conservation.^48^ Among the genes associated with inflammation-unique accessible chromatin regions, ∼17% (322 genes) encoded transcription factors, including FOS, JUN, ATF3, TCF7L2, SOX4, and TGFB2, known regulators of inflammatory signaling and stem cell regulation (Figure 4*C* and Supplementary Figure 5*A*). We next cross-referenced inflammation-unique regions with curated transcription factor target gene sets from the MSigDB Hallmark collection and found significant enrichment for targets of inflammatory transcription factors such as RELA (NF-κB), NFKB1, IRF8, STAT3, and TCF4 (Figure 4*D*), reinforcing the idea that inflammation leaves ISCs epigenetically primed at key regulatory hubs.

To this point, our analysis focused on genes linked to I-unique open chromatin, revealing a regulatory landscape that is epigenetically accessible but largely transcriptionally inactive. To better understand the broader transcriptional shifts that may contribute to the I phenotype, we next examined genome-wide expression differences between I and NI organoids. Pairwise correlation analysis of gene expression showed that organoids from the same patient (I and NI) were more similar to each other than to organoids from other patients, regardless of inflammation status, indicating patient-specific transcriptional identity is maintained (Supplementary Figure 5*B*). Genes upregulated in I organoids included IGFBP1, PRAC1/2, and BMP6, factors associated with stress responses, epithelial remodeling, and altered signal transduction, and downregulated genes such as PITX2, GATA5, and HOXC10, are developmental transcription factors often associated with tissue specification, differentiation, and epithelial identity (Supplementary Figure 5*C*). Gene set enrichment analysis (GSEA) on the full transcriptome showed that PI organoids were enriched for genes downregulated by interleukin (IL)-6 deprivation, epidermal growth factor receptor (EGFR) signaling, glycolysis, and proliferative cycling (Supplementary Figure 5*D*), further supporting a shift toward a quiescent or stress-adapted transcriptional state.

Our findings reveal 2 distinct layers of transcriptional regulation in inflammation-experienced ISCs. Whole transcriptomic analysis revealed I organoids exhibited elevated stress- and epithelial remodeling-associated programs and reduced expression of differentiation factors, features that may underlie the impaired regeneration and barrier dysfunction. In parallel, the genes associated with inflammation-unique open chromatin regions were not transcriptionally upregulated. Given their enhancer-like features and enrichment for key transcription factor motifs, we hypothesized that these regions may represent regulatory elements primed for rapid activation upon challenge. Thus, we next asked whether inflammation-unique accessible genes mediate a heightened transcriptional response following inflammatory stimulus.

### Prior Inflammation Enhances ISC Response to Secondary Inflammatory Challenge

**Figure 5:**
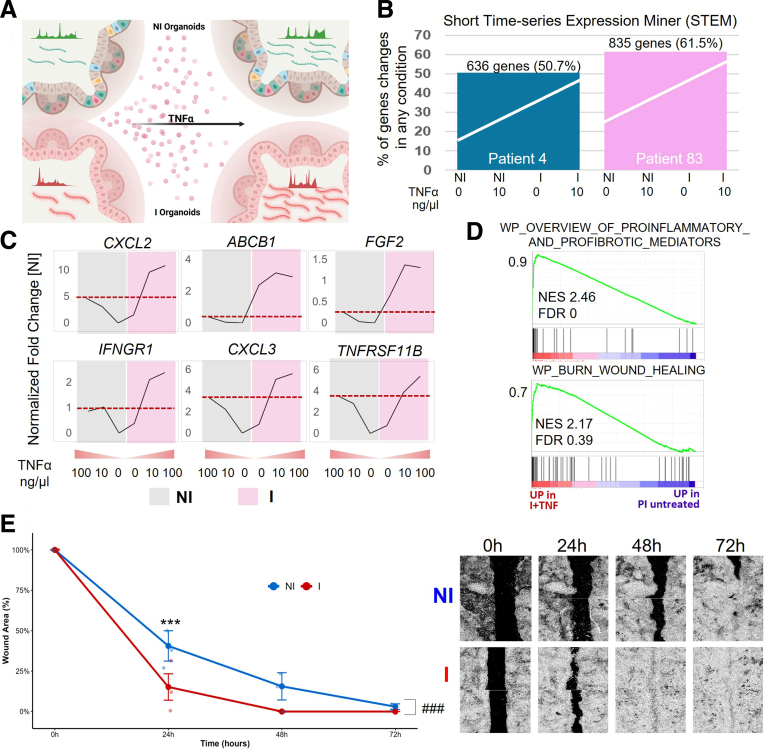
**I organoids exhibit enhanced transcriptional responses to inflammatory rechallenge.** (*A*) Experimental design of TNFα re-stimulation in NI and I organoids. (*B*) STEM analysis indicates a higher percentage of transcriptionally responsive genes in I vs NI organoids. (*C*) Expression profiles of representative TNFα-responsive genes across increasing TNFα concentrations (0, 10, 100 ng/μL) in I (*pink*) and NI (*gray*) organoids. Genes such as CXCL2 and CXCL3 showed enhanced induction in I organoids. (*D*) GSEA revealed increased activation of pathways related to proinflammatory mediators (*top*) and wound healing (*bottom*) in I organoids after TNFα treatment. (*E*) Time-dependent changes in wound area indicate that I organoid-derived monolayers heal faster than NI organoid-derived monolayers over 72 hours of culture (n = 3 patients). Data represent mean ± SEM. ∗∗∗*P* < .001 vs NI; ^###^*P* < .001 (time × condition). LMM with fixed factors: condition and time; random factor: patient. *Left*: Quantification of wound closure over 3 days. *Right*: Representative images of wound closure across time points.

The open chromatin regions identified in I organoids raised the possibility that prior inflammation had imprinted an epigenetic memory of a primed transcriptional state in ISCs. We hypothesized that these open chromatin regions function to endow the cell with the ability to respond rapidly to a secondary inflammatory challenge. To functionally test this, we rechallenged matched I and NI organoids with tumor necrosis factor α (TNFα) and performed 2 sets of RNA-seq experiments to evaluate gene expression changes (Figure 5*A*). TNFα was selected as the inflammatory stimulus given that TNFα is a well-characterized upstream activator of AP-1 and has been previously used to model inflammatory signaling in intestinal organoids derived from patients with IBD.^49^ We performed a dose-response RNA-seq experiment in organoids from patient 4, treating NI and I organoids with 10 ng/μL and 100 ng/μL TNFα, which revealed a broader transcriptional response in I organoids across both concentrations (Supplementary Figure 6*A* and *B*). Based on these findings, we proceeded with RNA-seq in organoids from 2 patients (Patients 4 and 83) treated with 10 ng/μL TNFα.

To assess dominant transcriptional responses to TNFα in NI and I organoids, we clustered differentially expressed genes (DEGs) based on shared expression trajectories using Short Time-series Expression Miner (STEM).^50^ In both patients, the most significantly enriched cluster represented genes more strongly induced in I organoids following TNFα treatment, comprising 50.7% and 61.5% of I-unique genes with open chromatin, indicating a broader transcriptional response in inflammation-experienced epithelium (Figure 5*B*, Supplementary Figure 7*A* and *B*). Analysis of representative inflammation-unique genes with open chromatin revealed more robust upregulation in the I organoids compared with NI in both experiments (Figure 5*C*; Supplementary Figure 6*C*). GSEA further demonstrated that the genes that were more robustly reactivated in the I organoids were enriched for proinflammatory and profibrotic gene programs and pathways linked to wound healing (Figure 5*D*). To evaluate whether this transcriptional memory impacted epithelial function, we performed a scratch assay in monolayers derived from NI and I organoids from 3 patients. I organoids exhibited significantly enhanced closure capacity compared with NI organoids with significance reached at 24 hours, across 3 days (Figure 5*E* and Supplementary Figure 8). This suggests that inflammation-experienced ISCs are functionally primed to mount an amplified regenerative response upon injury. Taken together, these findings indicate that ISCs do not return to a naïve state after inflammation but instead retain a molecular memory that shapes future cellular responses to inflammatory injury.

## Discussion

**Figure 6:**
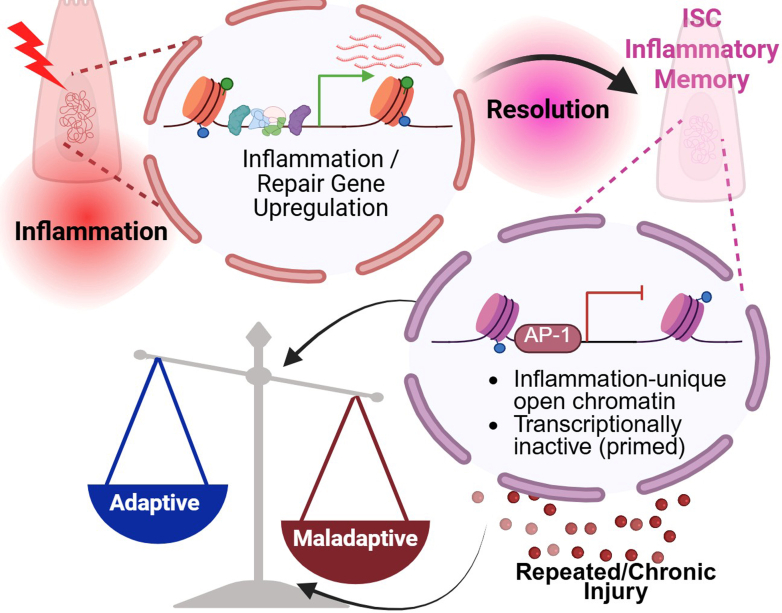
**Model of inflammatory memory in ISCs.** Inflammation induces chromatin remodeling in ISCs at enhancer regions linked to genes involved in inflammation and tissue repair. After inflammation resolves, a subset of regions remains open, establishing a primed chromatin state characteristic of inflammatory memory. This memory endows ISCs with enhanced transcriptional responsiveness upon secondary inflammatory challenge. Although this primed state can support adaptive responses, such as rapid reactivation of gene programs and healing following rechallenge, it may also predispose to maladaptive outcomes under conditions of repeated or chronic injury.

This study uncovers a previously unrecognized layer of stem cell regulation in IBD, by providing direct evidence that the intestinal epithelium retains a durable epigenetic memory of prior inflammation. Although prior studies have shown that IBD-derived organoids retain features of epithelial dysfunction,^31^, ^32^, ^33^, ^34^ none have interrogated chromatin alterations in matched inflamed and uninflamed regions from the same patient.^35^, ^36^, ^37^ Using paired human colonic organoids derived from inflamed and uninflamed regions of the same patients with UC, we demonstrate that prior inflammatory exposure leaves a stable chromatin imprint in ISCs, characterized by sustained accessibility at enhancer-like regulatory elements, enrichment for AP-1 transcription factor motifs, and enhanced transcriptional responsiveness to subsequent inflammatory stimuli (Figure 6, working model). Because organoid cultures are initiated and maintained by ISCs, persistent epigenetic features must reflect stem cell-intrinsic memory mechanisms. These findings establish that ISCs are not merely reset to a naïve state following resolution of inflammation but instead maintain a molecular memory that can impact future cellular behavior. Future work using single-cell chromatin and transcriptomic profiling will be necessary to confirm ISC-specific memory signatures and determine how these epigenetic programs are propagated into progeny lineages during epithelial renewal.

The persistent chromatin accessibility observed in inflammation-experienced organoids was concentrated at enhancer-like regions near genes involved in epithelial stress responses, regeneration, and inflammatory signaling. Although many of these regions were transcriptionally silent under homeostatic conditions, challenge with TNFα showed enhanced transcriptional activation in PI organoids compared with NI organoids. These data support the interpretation that inflammation-experienced ISCs exist in a transcriptionally primed state defined as silent under homeostasis but rapidly inducible upon secondary challenge.

This memory may serve an adaptive function, enabling ISCs to respond more efficiently to recurrent insults and support rapid epithelial regeneration. Indeed, PI organoids displayed enhanced wound healing responses compared with NI organoids at baseline and when re-exposed to inflammatory stimuli, suggesting that prior inflammatory exposure may prime ISCs for accelerated repair. However, our findings also raise the possibility that this same epigenetic imprinting may become maladaptive, predisposing the epithelium to dysregulated repair, persistent activation of inflammatory pathways, or eventual progression to neoplasia. In the context of UC, where disease recurrence often arises at previously affected sites despite histologic healing,^51^ such persistent reprogramming of ISCs may contribute to the chronic and relapsing nature of the disease. Whether inflammatory memory in ISCs influences disease recurrence remains an open and critical question.

This work highlights the power of human organoid systems for uncovering stable, cell-intrinsic features of disease that are not captured by short-term transcriptomic profiling alone. Although further studies are needed to define how inflammatory memory evolves over time and whether it can be erased or reprogrammed, our findings provide a foundation for understanding how ISCs integrate past inflammatory experience into future behavior. Targeting memory-associated transcription factors or chromatin remodelers may offer novel therapeutic strategies to modulate epithelial responses and promote mucosal healing in patients with chronic disease. This opens the door to new therapeutic strategies aimed at modulating the epigenetic landscape of ISCs to either enhance regenerative responses in acute inflammation or dampen maladaptive programs in chronic disease. Understanding how to balance the protective vs pathogenic aspects of inflammatory memory will be critical for designing interventions that promote long-term mucosal healing without fueling inflammation-associated complications.

In summary, we demonstrate that that inflammation leaves a lasting molecular imprint in ISCs from patients with UC that shapes their future responses to injury. This memory is encoded in accessible chromatin landscapes that potentially primes cells for enhanced transcriptional and functional responses to subsequent insult. These findings provide a foundational framework for understanding how stem cells integrate past inflammatory exposures into future tissue behavior, an insight that may help reframe epithelial resilience and vulnerability in chronic diseases such as IBD. Inflammatory memory in the intestinal epithelium may represent a double-edged sword: supporting regenerative readiness on one hand, while perpetuating disease progression on the other. Understanding and modulating this memory may open new avenues for promoting durable healing and preventing relapses in patients with IBD.

## Methods

### Sex as a Biological Variable

Both male and female participants were included equally in this study. Colonic tissue biopsies were obtained from patients with UC of both sexes undergoing endoscopy. Due to the paired, within-patient design, statistical comparisons of sex effects were not performed, and the study was not powered for sex-based subgroup analyses.

### Human Organoid Generation and Culture

All patient-derived organoids used in this study were collected from Mayo Clinic patients following informed consent (please see “Study Approval” section below). Information on tissue location and macroscopic inflammation status was registered at the time of sample collection. Organoids were generated following the isolation of crypts from fresh tissues collected during biopsy or surgery using established procedures.^26^^,^^28^ Briefly, crypts were released from freshly collected tissue using 5 mM ethylenediaminetetraacetic acid (EDTA), rocking at 4°C for 60 to 75 minutes. Isolated crypts were then embedded in ice-cold Matrigel (Corning Matrigel Growth Factor Reduced Product #356231), plated in 24-well plates, and overlaid with Human Colon Media (ADMEM containing 50% Wnt, R-Spondin, and Noggin [WRN] from conditioned media of L-WRN cell line [ATCC] and supplemented with: 1× N2 supplement, 1× B27 supplement, 40 ng/mL EGF, 3 uM SB202190, 500 nM A 83-01, 10 uM Y-27632, 1 uM NAC, 10 mM Nicotinamide, 10 nM Gastin I, 100 ug/mL Primocin, 1× antibiotic/anti-mycotic). For passaging, organoids were collected and digested in TrypLE Express (Gibco, #12604013) until small fragments or single cells were produced, which were counted. Media was changed every 3 days.

### OFE and Morphology

Each line was seeded at a standardized density of 50,000 cells to ensure comparable starting conditions. Morphological assessment, OFE, and FITC-dextran permeability assays were performed on the same organoid cultures, allowing for integrated assessment of multiple phenotypic parameters under identical experimental conditions. Organoids were dissociated into single cells using TrypLE Express (Gibco, #12604013) and were strained using a Falcon 40-uM cell strainer (Corning, #352240). The single-cell suspension was counted on a hemocytometer and Countess II Automated Cell Counter (Thermo Fisher). The single cells were plated in 8-ul domes of 100% Matrigel (Corning, #35623) in a 96-well flat-bottom cell culture plate (Starstedt, #83.3924.500). After plating, 100 ul of Human Colon Media was added to each well, and 50 ul of Human Colon Media was added to each well every 2 to 3 days. Morphology was monitored over an 8-day culture period across 3 consecutive passages. Brightfield images were acquired on days 2, 4, 6, and 8 using an EVOS imaging system. Surface area (μm^2^) was quantified from 50 organoids per timepoint using ImageJ software. Organoid-forming efficiency was assessed on day 4 of each passage by imaging 3 replicate wells and manually counting organoids using the Cell Counter plugin in ImageJ.

### Seeding Organoids as Monolayers on Transwell Cell Culture Inserts

A 2% Matrigel solution was made by diluting Matrigel in phosphate-buffered saline (PBS) (Corning, #35623). Two hundred uL of the 2% Matrigel solution was placed on the upper (apical) reservoir in a 24-well plate with a Transwell cell culture insert with a 3.0-uM pore size (Corning, #CLS3415) for 2 to 3 hours in an incubator at 37°C. Organoids were dissociated into single cells as described above. The 2% Matrigel solution was removed, and 3 × 10^5^ cells of the single cell suspension were placed in the apical reservoir on the Transwell insert. The total volume added to the apical reservoir with the single-cell suspension was 200 uL, and 600 uL of our organoid medium was added to the lower (basal) reservoir. Media was changed every 2 to 3 days until a monolayer covered the insert of the Transwell.

### Assessment of Barrier Function in NI and PI Organoid Monolayers (TER and FITC-Dextran)

Initially, TER was measured manually using an epithelial voltohmmeter 2 which was connected to a STX2 chopstick electrode (World Precision Instruments). This was done to ensure a confluent monolayer prior to beginning TER measurements. The Transwells were moved to a 24-well cell module cellZscope system (NanoAnalytics) with 1 mL and 200 μL of organoid media added to the basolateral and apical chambers, respectively. This real-time recording system allows for parallel impedance spectroscopy measurements of monolayers grown on semi-permeable membranes. The cellZscope module and organoid monolayers were housed in a humidified incubator (37°C and 5% CO_2_) for the duration of experimentation, with TER measurements taken every hour. Continuous TER measurements were recorded with raw TER data extracted and reported as TER measured over time. All experiments involving organoid monolayers were done using 6 biological replicates from each location (NI or PI) to reduce any monolayer-to-monolayer variability. Data was subsequently reported as an average for that location with each data point plotted representing those 6 monolayers per group. Analysis and calculations were performed in GraphPad Prism using analysis of variance (ANOVA).

For FITC-dextran permeability assessment, organoids were carefully extracted from Matrigel into microcentrifuge tubes using cutoff pipette tips to minimize mechanical stress. Organoids were then incubated with (FITC)-conjugated dextran (4 kDa, Sigma-Aldrich) at a concentration of 1 mg/mL for 30 minutes at room temperature in the dark. Following incubation, organoids were washed 3 times with PBS and mounted on glass slides for imaging. Fluorescent imaging was performed using an EVOS microscope with GFP filter settings at fixed light intensity. Only organoids with clearly visible lumens were selected for analysis. Approximately 15 to 25 organoids were analyzed per condition. For quantification, the outer boundary of each organoid was traced on the brightfield image to define a region of interest (ROI). This ROI was then applied to the corresponding fluorescent image to measure FITC fluorescence within the organoid. Corrected total cell fluorescence (CTCF) was calculated as: integrated density − (organoid area × mean fluorescence of background readings). This calculation corrects for both autofluorescence and organoid size. Higher CTCF values indicate increased barrier permeability.

### Immunofluorescence Staining

Organoids were recovered from Matrigel and fixed with 4% formaldehyde solution for 30 minutes at room temperature. Following fixation, organoids were permeabilized and blocked for 2 hours at room temperature using 10% goat serum and 0.3% Triton X-100. Organoids were then incubated overnight at 4°C with primary antibodies: rabbit anti-Ki67 (1:250, Abcam, AB16667) or rabbit anti-ZO-1 (1:250, Proteintech, 21773-1-AP). After washing, organoids were incubated for 2 hours with the corresponding secondary antibodies: Alexa Fluor 488 goat anti-rabbit (1:800, Invitrogen, A32731) for Ki67 or Alexa Fluor 594 goat anti-rabbit (1:800, Abcam, AB150080) for ZO-1. Nuclei were counterstained with 4′,6-diamidino-2-phenylindole (DAPI; 2.5 mg/mL, Invitrogen, 62248). Organoids were mounted on glass slides using ProLong Diamond Antifade Mountant (Invitrogen, P36961) and imaged using a Leica confocal microscope.

For Ki67 proliferation analysis, the number of Ki67-positive cells was quantified relative to the total organoid surface area, determined by DAPI staining. A linear mixed model was fitted with condition (NI vs I) as a fixed effect and patient as a random effect to account for interpatient variability. For ZO-1 analysis, organoid ROIs were defined from the DAPI channel using automated masking, and ZO-1 fluorescence intensity was quantified as CTCF per unit area, calculated by subtracting the mean background intensity from the mean ZO-1 intensity within the organoid mask. ZO-1 junction integrity was further assessed by segmenting junctions using a fixed absolute intensity threshold calibrated from the NI organoid images, followed by skeletonization to quantify total junction length and junction coverage (skeleton length normalized to organoid area). A fixed threshold was applied uniformly across all images to ensure that differences in junction detection reflect true differences in ZO-1 signal rather than adaptive contrast adjustments. Group comparisons (NI vs I) were performed using paired *t*-tests.

### ATAC-Seq in Organoids

Single-cell suspensions of patient-derived organoids were prepared by trypsinization using TrypLE Express (Gibco, #12604013) until dissociation was achieved and followed by gentle mechanical disruption and strained with a 40-uM strainer (Falcon, #352340) and counted on the Thermo Fisher Countess II automated cell counter. ATAC-seq was performed according to Buenrostro et al^52^ and Corces et al.^53^ Briefly, 50,000 cells were harvested and incubated at 4°C for 15 minutes in ATAC resuspension buffer (10mM Tris-HCl pH 7.4, 10mM NaCl, 3mM MgCl_2_, 0.1% NP-40, 0.1% Tween-20, 0.01% digitonin). Nuclei were washed with the ATAC resuspension buffer without detergents followed by centrifugation at 500 × g for 10 minutes at 4°C. TDE1 enzyme (Illumina, cat no. 20034198) was used for tagmentation for 30 minutes at 37°C in 1X Tagment DNA Buffer, 0.02% Digitonin (Promega, cat no. G9441), 0.2% Tween-20. DNA was extracted using MinElute kit (Qiagen) and amplified using NEBNext High-Fidelity PCR Master Mix (NEB). DNA fragment selection was performed using sparQ PureMag Beads (Quanta bio, cat no. 95196). Quality of DNA was evaluated using the High Sensitivity D1000 ScreenTape (Agilent) run on a Tapestation 4150 (Agilent). Next-generation sequencing was performed on NextSeq 2000 PE50 in the Genome Analysis Core in Mayo Clinic, Rochester. See Supplementary Methods for details on bioinformatic analysis for ATAC-seq.

### RNA-Seq in Organoids

The same cells from organoids prepared for ATAC-seq were harvested by centrifugation and the pellet used for RNA extraction. RNA was extracted by miRNeasy Micro Kit (Qiagen) for total RNA extraction according to manufacturer’s instructions. RNA from treated organoids was extracted using miRNeasy Micro Kit (Qiagen) according to manufacturer’s instructions. Libraries were prepared using the stranded mRNA prep, ligation kit (Illumina) following the manual instructions. Quality of libraries was validated using Tapestation 4150 (Agilent). Next-generation sequencing was performed on NextSeq 2000 PE50 in the Genome Analysis Core in Mayo Clinic, Rochester. See Supplementary Methods for details on bioinformatic analysis for RNA-seq.

### Organoid Exposure to Inflammatory Stimuli

Organoids were seeded at equal cell densities and treated with TNFα (Gibco, #AF-300-01A), which was supplemented in our 50% WRN media at a final concentration of 10 and 100 ng/mL for 24 hours. Organoids were harvested by removing TNFα supplemented media and adding 500 uL of Cell Recovery Solution to each dome (Corning, #354253). Organoids were placed on ice for 30 minutes in Cell Recovery Solution to remove Matrigel. Organoids were then washed 2× with PBS, and RNA was extracted and RNA-seq performed as described above.

### Organoid Wound Healing Assay

Single cells dissociated from intestinal organoids were seeded onto IBIDI 24-well culture inserts (IBIDI Cat.No: 80242) pre-coated with 2% Matrigel. Monolayers were cultured in organoid growth media and allowed to proliferate until they reached approximately 95% confluency, at which point the inserts were removed, creating 2 wound gaps per patient line. The culture plates were then placed into the IncuCyte live-cell imaging system, which was used to automatically capture phase-contrast images at regular intervals over 72 hours. Wound closure was monitored at designated time points (0, 24, 48, and 72 hours). The wound area at 0 hours was defined as 100% open, and closure was expressed as the percentage reduction in wound area relative to this baseline at each subsequent time point. Wound area quantification was performed using ImageJ.

### Statistical Analyses

Statistical analyses were performed using GraphPad Prism 8.0.1 (GraphPad Software, Inc), and R (version 4.5.1). Linear mixed-effects models (LMMs) were used to account for repeated measurements from the same patients across multiple passages, with patient included as a random effect. For organoid formation efficiency and barrier function, models included condition (NI vs I) and passage (P1, P2, P3) as fixed effects. For surface area growth kinetics, condition, time (day 2, 4, 6, 8), and passage were included as fixed effects. For Ki67 proliferation, condition was included as a fixed effect. For wound healing, a LMM fitted with condition (NI vs I), time, and their interaction as fixed effects and patient as a random effect to account for interpatient variability. Post-hoc pairwise comparisons were performed using estimated marginal means with Kenward-Roger approximation. Results are presented as mean ± standard error of the mean (SEM). Statistical significance was determined at α = 0.05. For RNA-seq and ATAC-seq data, differential analysis was performed using DESeq2 and DiffBind, respectively, with multiple hypothesis testing correction applied (Benjamini-Hochberg false discovery rate [FDR] < 0.05); more details can be found in the Supplementary Methods section. The number of biological replicates and specific statistical tests used are indicated in figure legends.

### Study Approval

This study was approved by the Mayo Clinic Institutional Review Board (IRB #21-006244). All human participants provided written informed consent prior to enrollment and tissue collection. All organoid derivation and experimental procedures were conducted in accordance with approved institutional review board protocols and institutional guidelines. No animal studies were performed.

## Supplementary Material

Supplementary Material

Extended PDF
