# Supplementary material for: Intestinal Stem Cells From Patients With Inflammatory Bowel Disease Retain an Epigenetic Memory of Inflammation
Source: Cell Mol Gastroenterol Hepatol. 2026 Mar 27;20(7):101774. doi: 10.1016/j.jcmgh.2026.101774 (PMC13196144; doi:10.1016/j.jcmgh.2026.101774)
Supplement: Supplementary Material [file mmc1.pdf]

SUPPLEMENTAL MATERIAL:

Supplementary Methods

Patient tissue processing, RNA extraction and qPCR for inflammatory assessment.

RNA was isolated from tissue using the Maxwell RSC SimplyRNA Tissue Kit (Promega, #AS1340) on the Maxwell RSC instrument (Promega). Total RNA was reverse-transcribed to cDNA using iScript Reverse Transcription Supermix (Bio-Rad, #1708441). Quantitative real-time PCR (qPCR) was performed on the CFX384 Real-Time System (Bio-Rad) with iTaq Universal SYBR Green Supermix (Bio-Rad, #1725121). Primer sequences (IDT) are listed in the table below. Raw Cq data were exported using CFX Maestro software (Bio-Rad). All qRT-PCR results were normalized to an inter-run calibrator to correct for run-to-run variation and to the geometric mean of three endogenous reference genes (*GAPDH, S18* and *ACTB*), which were confirmed to be unaffected by inflammation, following the method of Vandesompele et al.[1]

Gene expression for a five-gene inflammatory panel (*CXCL8, CXCL10, SAA1, IL1B, TNF*) was used to calculate the inflammatory score. For each gene, ΔΔCt values were transformed to z-scores by centering and scaling using the mean and standard deviation calculated from the non-inflamed (NI) samples for that gene. This same transformation was applied to the inflamed (I) samples, so that each I z-score reflects the deviation from the NI mean in NI standard-deviation units. A composite inflammatory score was computed for each sample as the mean of these z-scores across the five genes. The composite score was additionally centered so that the mean of the NI samples equaled zero. Composite scores were compared between matched tissues using a linear mixed-effects model fitted to gene-level z-scores with Tissue (NI vs I) as a fixed effect and random intercepts for Subject and Gene.

qPCR primer list. All primers were designed against *Homo sapiens* gene sequences.

ABBREVIATION	Forward Primer (5'-3')	Reverse Primer (5'-3')
GAPDH	GTCTCCTCTGACTTCAACAGCG	ACCACCCTGTTGCTGTAGCCAA
RPS18	ACCCGTTGAACCCCATTCGTGA	GCCTCACTAAACCATCCAATCGG

ACTB	CACCATTGGCAATGAGCGGTTC	AGGTCTTTGCGGATGTCCACGT
CXCL8	CCACCGGAAGGAACCATCTC	TTGGCAAAACTGCACCTTCAC
CXCL10	TCCACGTGTTGAGATCATTGCTA	CGATTTTGCTCCCCTCTGG
SAA1	CTACAGCACAGATCAGCACCA	TCCCCTTTTGGCAGCATCAT
IL1B	GCACGATGCACCTGTACGAT	ACCAAGCTTTTTTGCTGTGAGT
TNF	CCTCTCTCTAATCAGCCCTCTG	GAGGACCTGGGAGTAGATGAG

Bioinformatic analysis in patient-derived organoids

Quality of reads from fastq raw files was evaluated using fastqc (v0.11.9) to check for base quality scores, adapter contamination, and sequence duplication levels. Reads passing quality metrics were mapped to hg38 (GRCh38, GCA_000001405.15) using bwa (v0.7.17)[2] with parameters -n 10 -a 500 -o 10000 -N 10 -s. Only uniquely mapped, non-duplicated reads with mapping quality (MAPQ > 30) were retained using samtools (v1.18).[3] Narrow peaks were called using MACS2 (v2.0.10)[4] with parameters: -f BAM -g hs --keep-dup all -q 0.01. Peak lists were then filtered to remove blacklisted regions (hg38-blacklist.v2.bed). To compare and visualize peak intensities across samples and conditions, normalized bigwig (bw) files were generated using the bamCoverage tool in deeptools (v3.5.1).[5] UC-specific regions were defined using the Bioconductor R package (Diffbind).[6] Differential accessibility analysis between inflamed and uninflamed groups was conducted using DiffBind (v2) in R (v4.2.2), applying edgeR for statistical modeling using default parameters and minMembers=3. Differentially accessible regions with a pvalue < 0.05 and |log2 fold change| > 1 were retained for downstream analysis. Deeptools (v3.5.1) was used to generate heatmaps and binding profiles. Associated genes with regions were defined using GREAT analysis.[7] Association rule used was Basal+extension: 5000 bp upstream, 1000 bp downstream, 5Mb max extension. Transcription factor motifs in the differentially accessible regions were identified using HOMER's findMotifsGenome.pl utility using default parameters.[8] To further analyze transcription factor binding at differentially open chromatin regions, TOBIAS (v0.13.3)[9] was used for footprinting. BAM files from all replicates were merged for each condition before footprinting analysis. TOBIAS was run with default

parameters for bias correction (ATACorrect), footprinting score calculation (ScoreBigwig), and transcription factor binding activity prediction (BINDetect). The JASPAR human core motif database was used as the motif set for TOBIAS BINDetect. Genes associated with differentially enriched peaks were analyzed for pathway enrichment using Enrichr web-based interface.[10–12] To identify potential upstream regulators of differentially enriched genes, CistromeDB Toolkit[13,14] and ChIP-Atlas[15] were used. Gene lists derived from differential peak analysis were submitted to CistromeDB, which provided rankings of candidate transcriptional regulators based on their binding enrichment. Additionally, ChIP-Atlas was used to cross-reference gene targets with publicly available ChIP-Seq datasets, further confirming key regulators. Genome-wide signal tracks were visualized in IGV (v2.3.93).[16,17] All analyses were conducted in a Linux-based computing environment running R (v4.2.2), Python (v3.10.7), and Java (JDK 13.0.2). Custom scripts for additional processing steps are available upon request.

Bioinformatic analysis for RNA-seq in patient-derived organoids

Raw paired-end sequencing reads were processed through the Mayo RNA-Seq bioinformatics pipeline, MAP-RSeq (v3.1.4).[18] Briefly, Fastqc (v0.11.8) was used to assess read quality and Fastp (0.20.0)[19] was used to trim adapter sequences. Trimmed reads were aligned to the hg38 reference genome using the splice-aware STAR aligner (v2.6.1c)[20] using strand-specific basic two-pass mapping. To detect chimeric transcripts, chimeric reads with minimum segment length of 12 nucleotides were retained, and junctions with minimum overhang of 12 nucleotides were reported. Additionally, the minimum overhang for annotated splice junctions was set to 10 nucleotides. The maximum intron length and gap between read mates was set to 200 kb. Aligned and unmapped reads were reported in the resulting coordinate-sorted BAM file. In addition, MultiQC (v1.14)[21] and RSEQC[22] were used for comprehensive quality control of the aligned reads. Gene-level expression quantification was performed using Subread featureCounts (v1.6.3)[23] with parameters -O -p -s 2 to obtain strand-specific raw gene counts

and normalized (FPKM – Fragments Per Kilobase per Million mapped reads) counts. Pairwise differential expression analyses were conducted with raw gene-level counts using edgeR (v3.40.2)[24] with default parameters. Genes were considered differentially expressed with Log2 Fold change >1 or < 1 and p-adjusted value < 0.05. To identify biologically enriched pathways, we used enrichR (v3.2)[10] and Gene Set Enrichment Analysis (GSEA)[25] got expressed genes using human gene set collections from the Molecular Signatures Database (MSigDB v2023.2.Hs) limiting to gene sets including 30-1000 genes.[26] Differently expressed genes were hierarchically clustered using Cluster 3.0[27] using Euclidean distance as a similarity metric and average linkage as a clustering method. Heatmaps were generated using Java Treeview 3.0.[28] Pathway enrichment analysis was performed in WEB-based GEne SeT AnaLysis Toolkit (Webgestalt)[29,30] using the following parameters: Over-Representation method, number analytes for category 30-2000, multiple test adjustment BH and a top 10 significance level. Differentially expressed transcription factors were used to build a TF-gene regulatory network using the TF-Target query of the iRegulon app[31] in Cytoscape 3.10.3.[32] To characterize gene signature behavior across TNF treatment, the short time-series expression miner (STEM)[33] was used with the following parameters: normalize data, STEM Clustering method, maximum number of model profiles set to 10 and maximum unit change in model profiles between points set to 3. Graphs were designed using GraphPad Prism 8.0.1 (GraphPad Software, Inc., San Diego, CA, USA), BioRender.com and R (4.0.3).

Supplemental References

1. Vandesompele J, De Preter K, Pattyn F, Poppe B, Van Roy N, De Paepe A, Speleman F. Accurate normalization of real-time quantitative RT-PCR data by geometric averaging of multiple internal control genes. Genome Biol 2002;3:RESEARCH0034.

2. Li H, Durbin R. Fast and accurate short read alignment with Burrows-Wheeler transform. Bioinformatics 2009;25:1754–1760.

3. Li H, Handsaker B, Wysoker A, Fennell T, Ruan J, Homer N, Marth G, Abecasis G, Durbin R, 1000 Genome Project Data Processing Subgroup. The Sequence Alignment/Map format and SAMtools. Bioinformatics 2009;25:2078–2079.

4. Zhang Y, Liu T, Meyer CA, Eeckhoute J, Johnson DS, Bernstein BE, Nusbaum C, Myers RM, Brown M, Li W, Liu XS. Model-based analysis of ChIP-Seq (MACS). Genome Biol 2008;9:R137.

5. Ramírez F, Ryan DP, Grüning B, Bhardwaj V, Kilpert F, Richter AS, Heyne S, Dündar F, Manke T. deepTools2: a next generation web server for deep-sequencing data analysis. Nucleic Acids Res 2016;44:W160-165.

6. Ross-Innes CS, Stark R, Teschendorff AE, Holmes KA, Ali HR, Dunning MJ, Brown GD, Gojis O, Ellis IO, Green AR, Ali S, Chin S-F, Palmieri C, Caldas C, Carroll JS. Differential oestrogen receptor binding is associated with clinical outcome in breast cancer. Nature 2012;481:389–393.

7. McLean CY, Bristor D, Hiller M, Clarke SL, Schaar BT, Lowe CB, Wenger AM, Bejerano G. GREAT improves functional interpretation of cis-regulatory regions. Nat Biotechnol 2010;28:495–501.

8. Heinz S, Benner C, Spann N, Bertolino E, Lin YC, Laslo P, Cheng JX, Murre C, Singh H, Glass CK. Simple combinations of lineage-determining transcription factors prime cis-regulatory elements required for macrophage and B cell identities. Mol Cell 2010;38:576–589.

9. Bentsen M, Goymann P, Schultheis H, Klee K, Petrova A, Wiegandt R, Fust A, Preussner J, Kuenne C, Braun T, Kim J, Looso M. ATAC-seq footprinting unravels kinetics of transcription factor binding during zygotic genome activation. Nat Commun 2020;11:4267.

10. Chen EY, Tan CM, Kou Y, Duan Q, Wang Z, Meirelles GV, Clark NR, Ma'ayan A. Enrichr: interactive and collaborative HTML5 gene list enrichment analysis tool. BMC Bioinformatics 2013;14:128.

11. Xie Z, Bailey A, Kuleshov MV, Clarke DJB, Evangelista JE, Jenkins SL, Lachmann A, Wojciechowicz ML, Kropiwnicki E, Jagodnik KM, Jeon M, Ma'ayan A. Gene Set Knowledge Discovery with Enrichr. Curr Protoc 2021;1:e90.

12. Kuleshov MV, Jones MR, Rouillard AD, Fernandez NF, Duan Q, Wang Z, Koplev S, Jenkins SL, Jagodnik KM, Lachmann A, McDermott MG, Monteiro CD, Gundersen GW, Ma'ayan A. Enrichr: a comprehensive gene set enrichment analysis web server 2016 update. Nucleic Acids Res 2016;44:W90-97.

13. Mei S, Qin Q, Wu Q, Sun H, Zheng R, Zang C, Zhu M, Wu J, Shi X, Taing L, Liu T, Brown M, Meyer CA, Liu XS. Cistrome Data Browser: a data portal for ChIP-Seq and chromatin accessibility data in human and mouse. Nucleic Acids Res 2017;45:D658–D662.

14. Zheng R, Wan C, Mei S, Qin Q, Wu Q, Sun H, Chen C-H, Brown M, Zhang X, Meyer CA, Liu XS. Cistrome Data Browser: expanded datasets and new tools for gene regulatory analysis. Nucleic Acids Res 2019;47:D729–D735.

15. Zou Z, Ohta T, Miura F, Oki S. ChIP-Atlas 2021 update: a data-mining suite for exploring epigenomic landscapes by fully integrating ChIP-seq, ATAC-seq and Bisulfite-seq data. Nucleic Acids Res 2022;50:W175–W182.

16. Thorvaldsdóttir H, Robinson JT, Mesirov JP. Integrative Genomics Viewer (IGV): high-performance genomics data visualization and exploration. Brief Bioinform 2013;14:178–192.

17. Robinson JT, Thorvaldsdóttir H, Winckler W, Guttman M, Lander ES, Getz G, Mesirov JP. Integrative genomics viewer. Nat Biotechnol 2011;29:24–26.

18. Kalari KR, Nair AA, Bhavsar JD, O'Brien DR, Davila JI, Bockol MA, Nie J, Tang X, Baheti S, Doughty JB, Middha S, Sicotte H, Thompson AE, Asmann YW, Kocher J-PA. MAP-RSeq: Mayo Analysis Pipeline for RNA sequencing. BMC Bioinformatics 2014;15:224.

19. Chen S, Zhou Y, Chen Y, Gu J. fastp: an ultra-fast all-in-one FASTQ preprocessor. Bioinformatics 2018;34:i884–i890.

20. Dobin A, Davis CA, Schlesinger F, Drenkow J, Zaleski C, Jha S, Batut P, Chaisson M, Gingeras TR. STAR: ultrafast universal RNA-seq aligner. Bioinformatics 2013;29:15–21.

21. Ewels P, Magnusson M, Lundin S, Käller M. MultiQC: summarize analysis results for multiple tools and samples in a single report. Bioinformatics 2016;32:3047–3048.

22. Wang L, Wang S, Li W. RSeQC: quality control of RNA-seq experiments. Bioinformatics 2012;28:2184–2185.

23. Liao Y, Smyth GK, Shi W. The Subread aligner: fast, accurate and scalable read mapping by seed-and-vote. Nucleic Acids Res 2013;41:e108.

24. Robinson MD, McCarthy DJ, Smyth GK. edgeR: a Bioconductor package for differential expression analysis of digital gene expression data. Bioinformatics 2010;26:139–140.

25. Subramanian A, Tamayo P, Mootha VK, Mukherjee S, Ebert BL, Gillette MA, Paulovich A, Pomeroy SL, Golub TR, Lander ES, Mesirov JP. Gene set enrichment analysis: a knowledge-based approach for interpreting genome-wide expression profiles. Proc Natl Acad Sci U S A 2005;102:15545–15550.

26. Liberzon A, Birger C, Thorvaldsdóttir H, Ghandi M, Mesirov JP, Tamayo P. The Molecular Signatures Database (MSigDB) hallmark gene set collection. Cell Syst 2015;1:417–425.

27. de Hoon MJL, Imoto S, Nolan J, Miyano S. Open source clustering software. Bioinformatics 2004;20:1453–1454.

28. Saldanha AJ. Java Treeview--extensible visualization of microarray data. Bioinformatics 2004;20:3246–3248.

29. Elizarraras JM, Liao Y, Shi Z, Zhu Q, Pico AR, Zhang B. WebGestalt 2024: faster gene set analysis and new support for metabolomics and multi-omics. Nucleic Acids Res 2024;52:W415–W421.

30. Zhang B, Kirov S, Snoddy J. WebGestalt: an integrated system for exploring gene sets in various biological contexts. Nucleic Acids Res 2005;33:W741-748.

31. Janky R, Verfaillie A, Imrichová H, Van de Sande B, Standaert L, Christiaens V, Hulselmans G, Herten K, Naval Sanchez M, Potier D, Svetlichnyy D, Kalender Atak Z, Fiers M, Marine J-C, Aerts S. iRegulon: from a gene list to a gene regulatory network using large motif and track collections. PLoS Comput Biol 2014;10:e1003731.

32. Shannon P, Markiel A, Ozier O, Baliga NS, Wang JT, Ramage D, Amin N, Schwikowski B, Ideker T. Cytoscape: a software environment for integrated models of biomolecular interaction networks. Genome Res 2003;13:2498–2504.

33. Ernst J, Bar-Joseph Z. STEM: a tool for the analysis of short time series gene expression data. BMC Bioinformatics 2006;7:191.

Supplementary Table 1. Patient Clinical Data.

Patient ID	Sample ID	Age (y)	Sex	Race	Ethnicity	Disease duration (y)	Region	Mayo endoscopic subscore	Biopsy or Surgical Tissue	Medications at collection
IBD_83	IBD_83UC<br>IBD_83N	20-29	M	White	Not Hispanic	3.8	Right colon<br>Left colon	2<br>0	surgical	none
IBD_13	IBD_13N<br>IBD_13UC	40-49	M	White	Not Hispanic	0.8	Sigmoid colon<br>Rectum	0<br>1	biopsy	vedolizumab, mesalamine
IBD_3	IBD_3N<br>IBD_3UC	30-39	F	White	Not Hispanic	5.9	Right colon<br>Left colon	0<br>2	biopsy	mesalamine
IBD_115	IBD_115N<br>IBD_115UC	20-29	M	White	Not Hispanic	4	Right colon<br>Left colon/Rectum	0<br>3	surgical	ustekinumab
IBD_4	IBD_4N<br>IBD_4UC	30-39	F	White	Not Hispanic	3	Right colon<br>Left colon/Rectum	0<br>3	surgical	vedolizumab, azathioprine, prednisone
IBD_125	IBD_125N<br>IBD_125UC	30-39	F	White	Not Hispanic	10	Right colon<br>Left colon/Rectum	0<br>3	surgical	upadacitinib

Supplemental Table 1. Baseline demographic and clinical data for study participants. y-years; m-months

SUPPLEMENTARY FIGURES AND LEGENDS

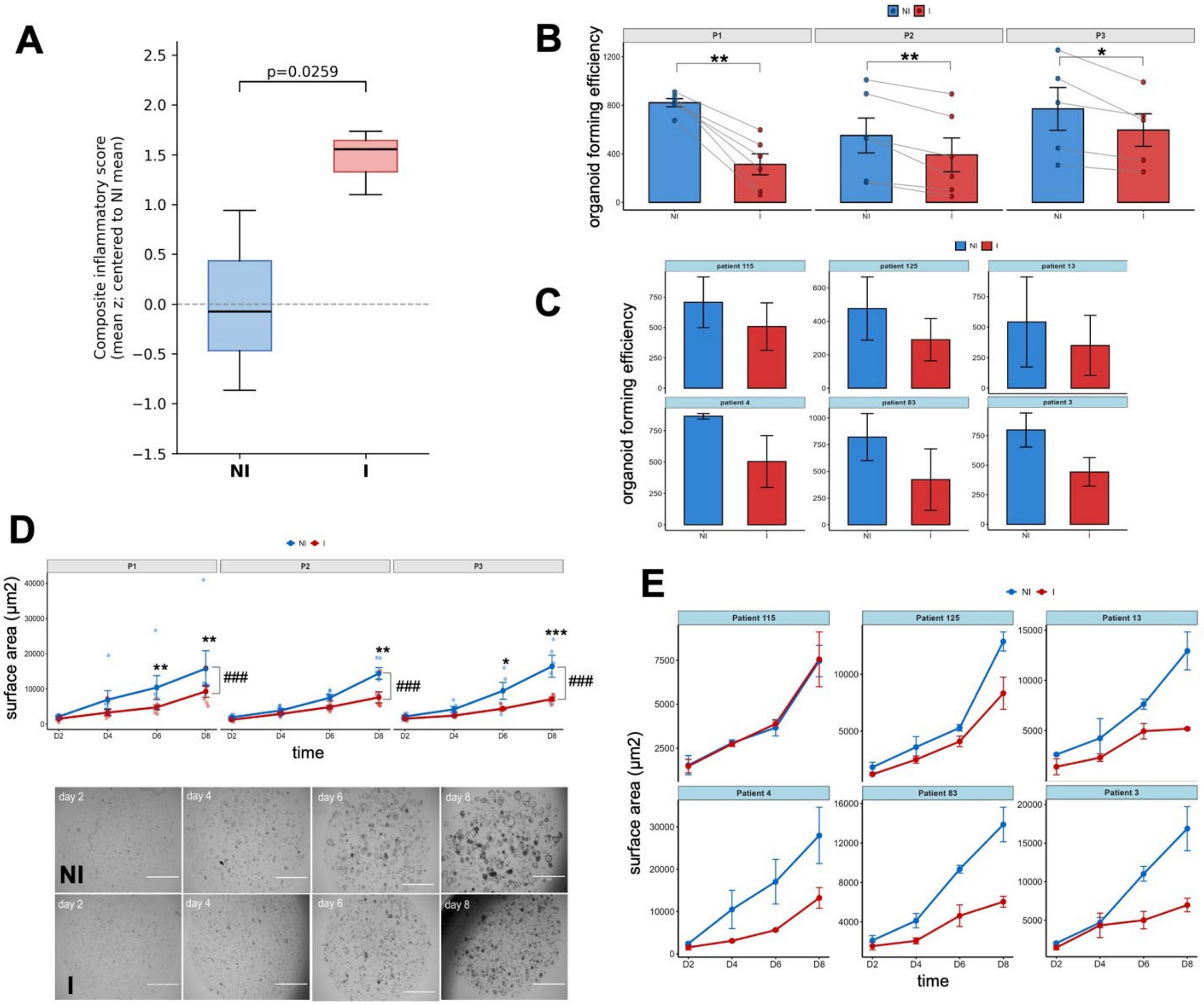

Figure S1. Passage- and patient-level data for NI and I organoid morphology. (A) A composite inflammatory score based on five pro-inflammatory genes (*CXCL8, CXCL10, SAA1, IL1B, TNF*) was calculated as the mean of NI-based gene z-scores (computed from raw ΔΔCt using the mean and SD of NI samples per gene) and centered so that the mean of NI samples equals zero. Box plots show median and interquartile range; whiskers extend to minimum and maximum values. P-value is from likelihood ratio test. (**B**) Organoid-forming efficiency (OFE) is reduced in I stem cells compared to NI stem cells across all three passages. Data represent the number of organoids generated from 50,000 predominantly undifferentiated crypt-based cells. Each panel shows one passage with data from all patients. (**C**) Patient-specific OFE

demonstrates consistent reduction in I colonoids compared to NI colonoids across all six patients. Each panel shows one patient with data from three passages. (**D**) Time-dependent changes in the surface area indicate that I colonoids grow slower than NI colonoids over 8 days of culture across all three passages. Left panel: Each panel shows one passage with data from all patients. Right panels show representative brightfield images for NI and I colonoids. scale bar = 200 μm. (**E**) Time-dependent changes in the surface area for individual patients show that I colonoids grow slower than NI colonoids in the majority of patients. Each panel shows one patient with data from three passages. For all panels, data represent mean ± SEM. *$p<0.05$, **$p < 0.01$, ***$p < 0.001$ vs. NI; ###$p < 0.001$ (time × condition). Linear mixed model with fixed factors: condition, passage, and time; random factor: patient.

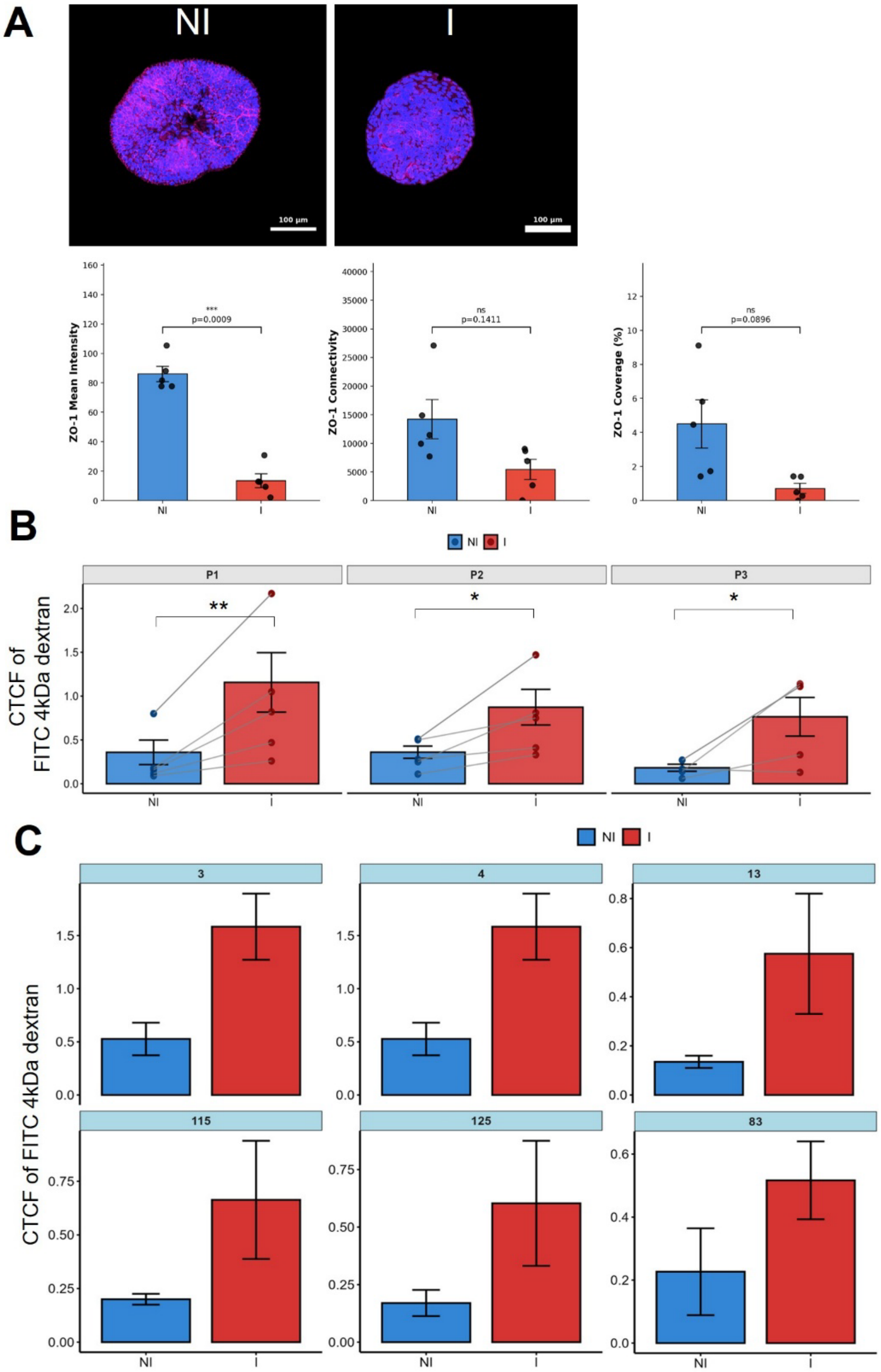

Figure S2. Passage- and patient-level data for NI and I barrier integrity. (A) Whole-mount immunofluorescence staining of ZO-1 in organoids reveals disrupted tight junctions in I compared to NI organoids. Representative images on the top show ZO-1 (pink) and nuclei

(DAPI, blue). Quantification of ZO-1 on bottom. (**B**) FITC-dextran (4 kDa) permeability is increased in I colonoids compared to NI colonoids across all three passages. Each panel shows one passage with data from all patients. Higher fluorescence quantified by corrected total cell fluorescence (CTCF) indicates greater paracellular permeability and weaker barrier function. (**C**) FITC-dextran (4 kDa) permeability quantified by corrected total cell fluorescence (CTCF) for individual patients demonstrates that I colonoids have increased permeability compared to NI colonoids. Each panel shows one patient with data pooled from three passages. For all panels, data represent mean ± SEM. *$p < 0.05$, **$p < 0.01$ vs. NI. Linear mixed model with fixed factors: condition, passage; random factor: patient.

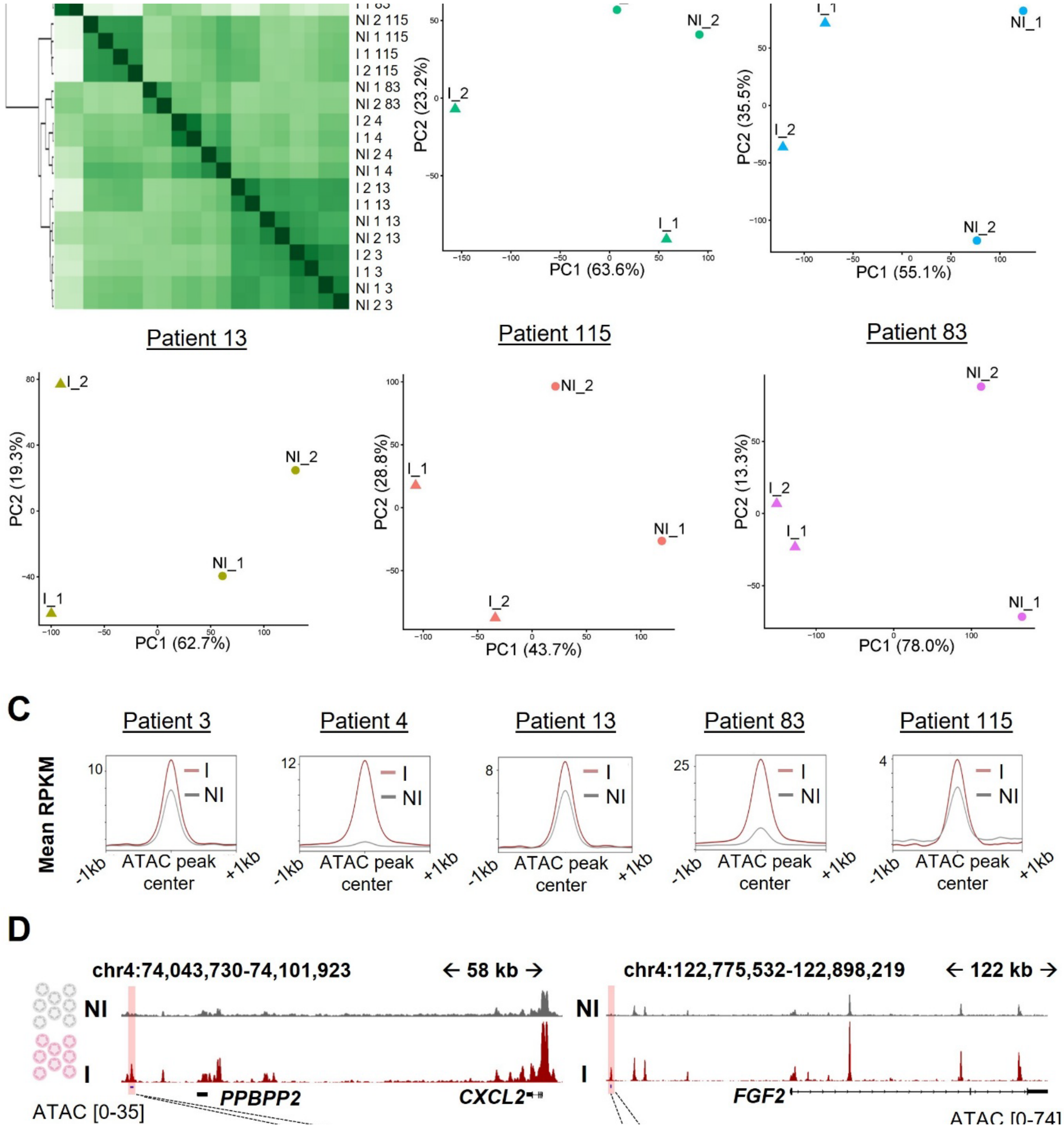

Figure S3. Chromatin Accessibility Patterns in Matched NI and I Organoids

(**A**) Sample-to-sample correlation heatmap showing high intra-patient similarity in ATAC-seq profiles across I and NI organoids. (**B**) Principal component analysis of ATAC-seq data for each plotted by patient (n=5) shows distinct separation of NI and I samples along PC1. (**C**) Mean signal plots of ATAC-seq signal centered on I-unique peaks. (**D**) Genome tracks showing increased accessibility at CXCL2 and FGF2 loci in I organoids.

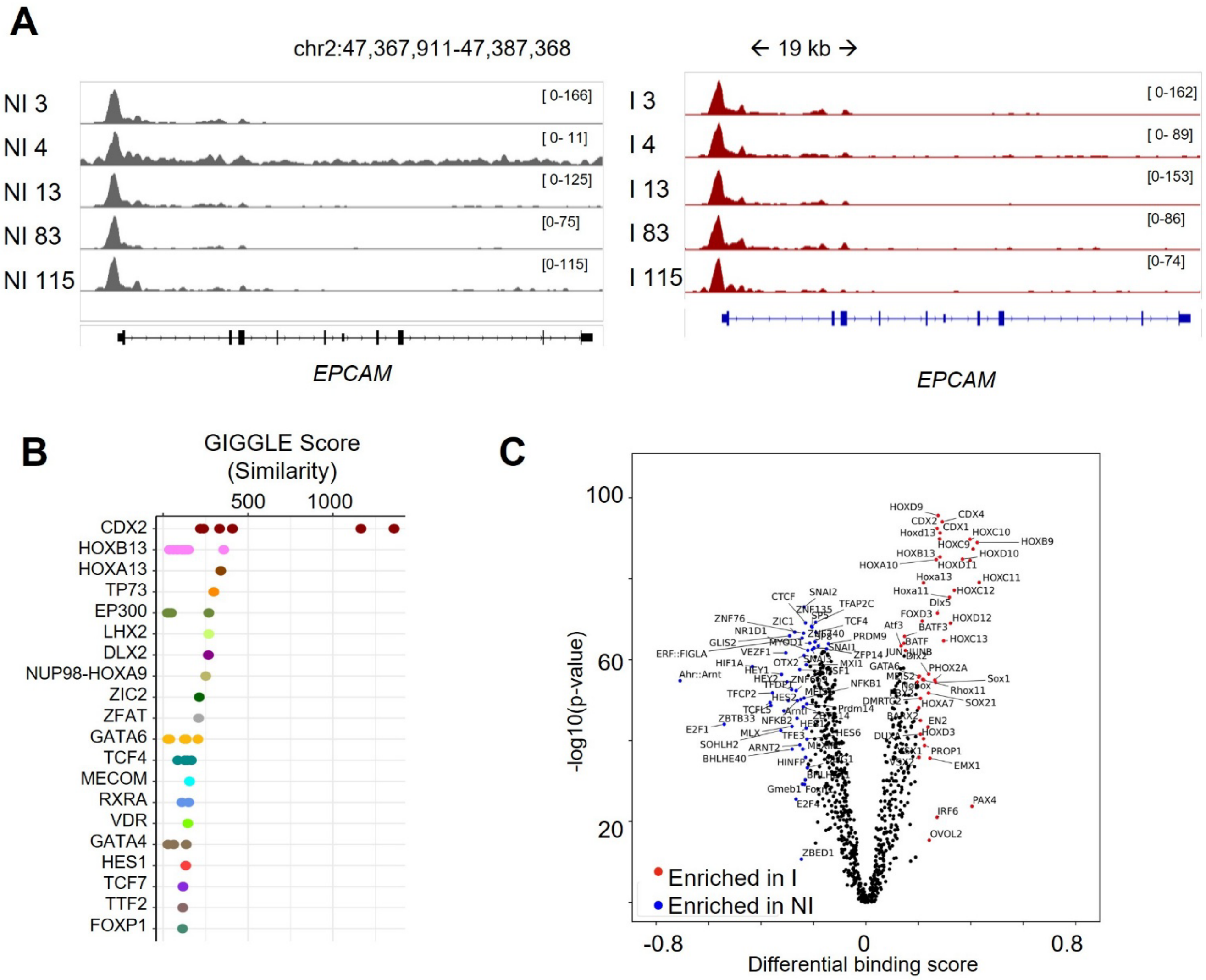

Figure S4. Inflamed Organoids Exhibit Conserved Epithelial Identity and Enrichment of Inflammatory and Lineage-Specific Motifs

(**A**) Genome browser tracks show conserved accessibility at the epithelial marker EPCAM in both NI and I organoids. (**B**) GIGGLE similarity analysis at inflammation-unique open chromatin. (**C**) Volcano plot of differential motif binding scores between I and NI organoids highlights motifs enriched in I (red) and NI (blue).

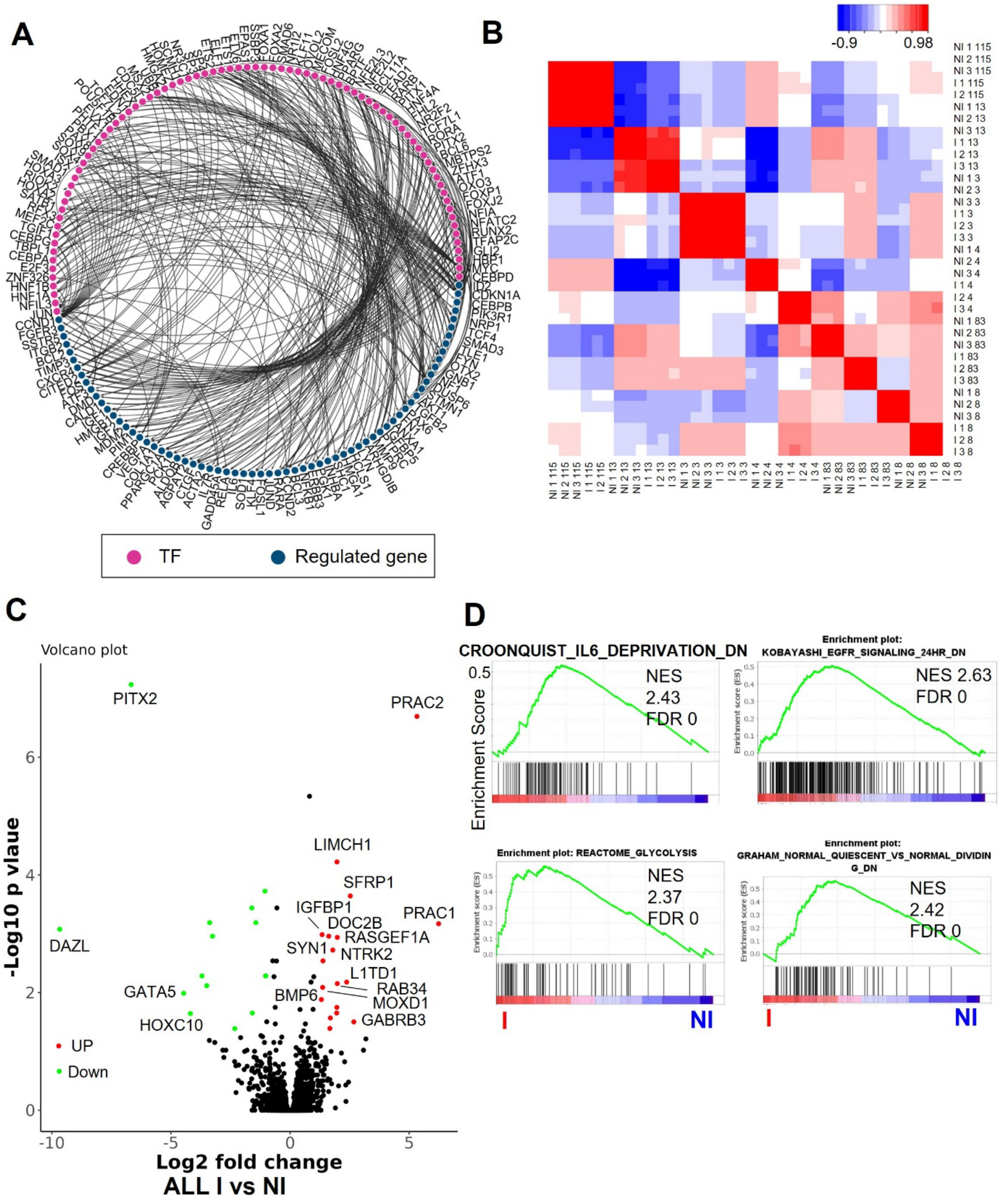

Figure S5. Differential Transcriptional Profiles and Regulatory Network Analysis

(**A**) TF-gene regulatory network from Figure 3C listed all TFs and regulated genes, only a subset are listed in the main figure. (**B**) Correlation matrix of RNA-seq profiles for each I and NI samples, in triplicate. (**C**) Volcano plot of differentially expressed genes between all I and NI

organoids. Upregulated genes in red; downregulated genes in green. (**D**) GSEA plots for all RNA-seq data.

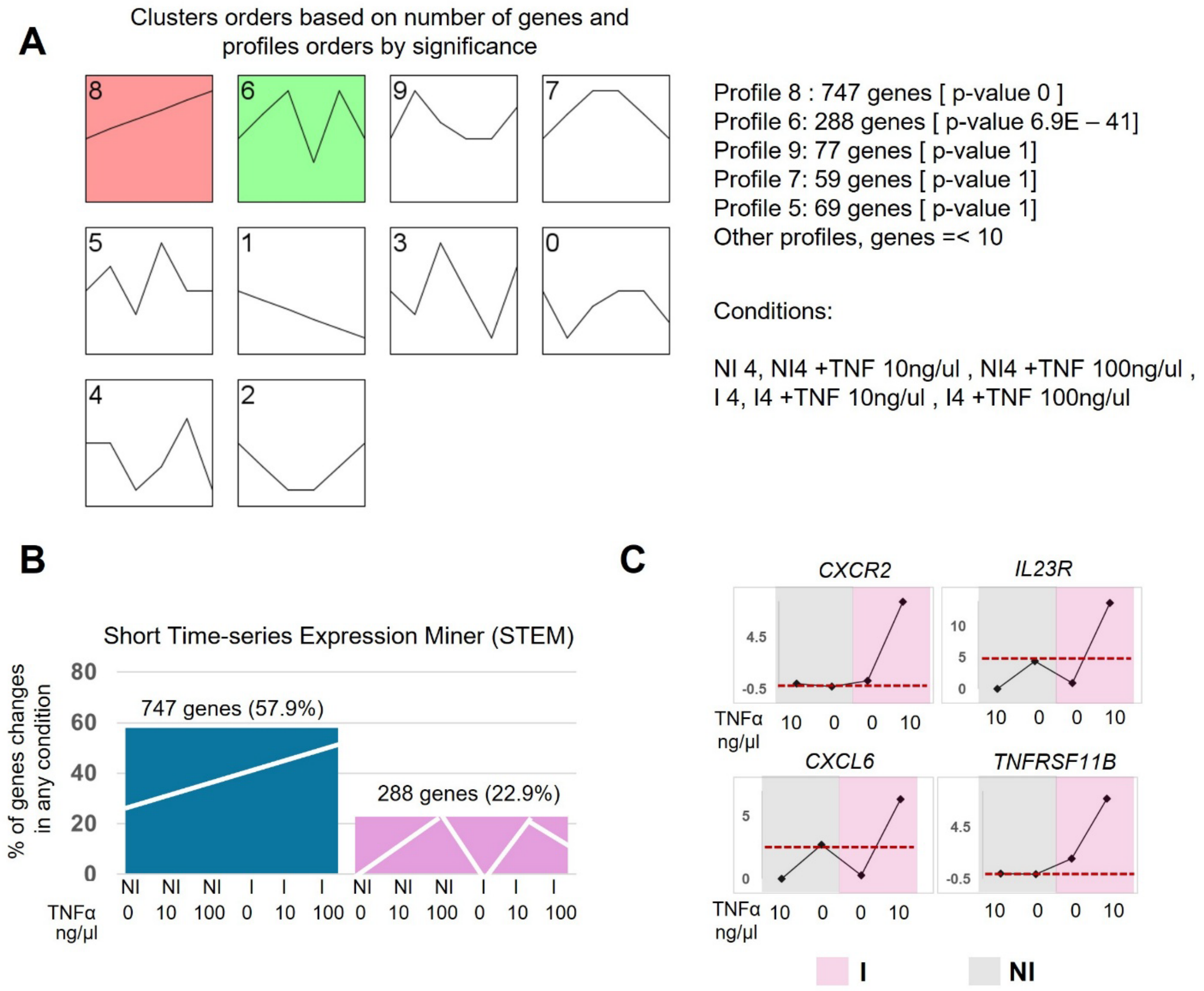

Figure S6. TNF-Induced Transcriptional Reactivation in Inflamed Organoids

(**A**) STEM analysis of I and NI organoids treated with 10 ng/uL or 100 ng/uL TNFα. Profile 8 (red highlighted) captures genes specifically reactivated in I organoids. (**B**) Summary of STEM analysis shows that 747 genes (57.9%) show this pattern of enhanced expression in I organoids treated with TNFα, while 22.9% of genes are reactivated by TNFα in both NI and I. (**C**) Line plots of representative genes (CXCR2, IL23R, CXCL6, and TNFRSF11B).

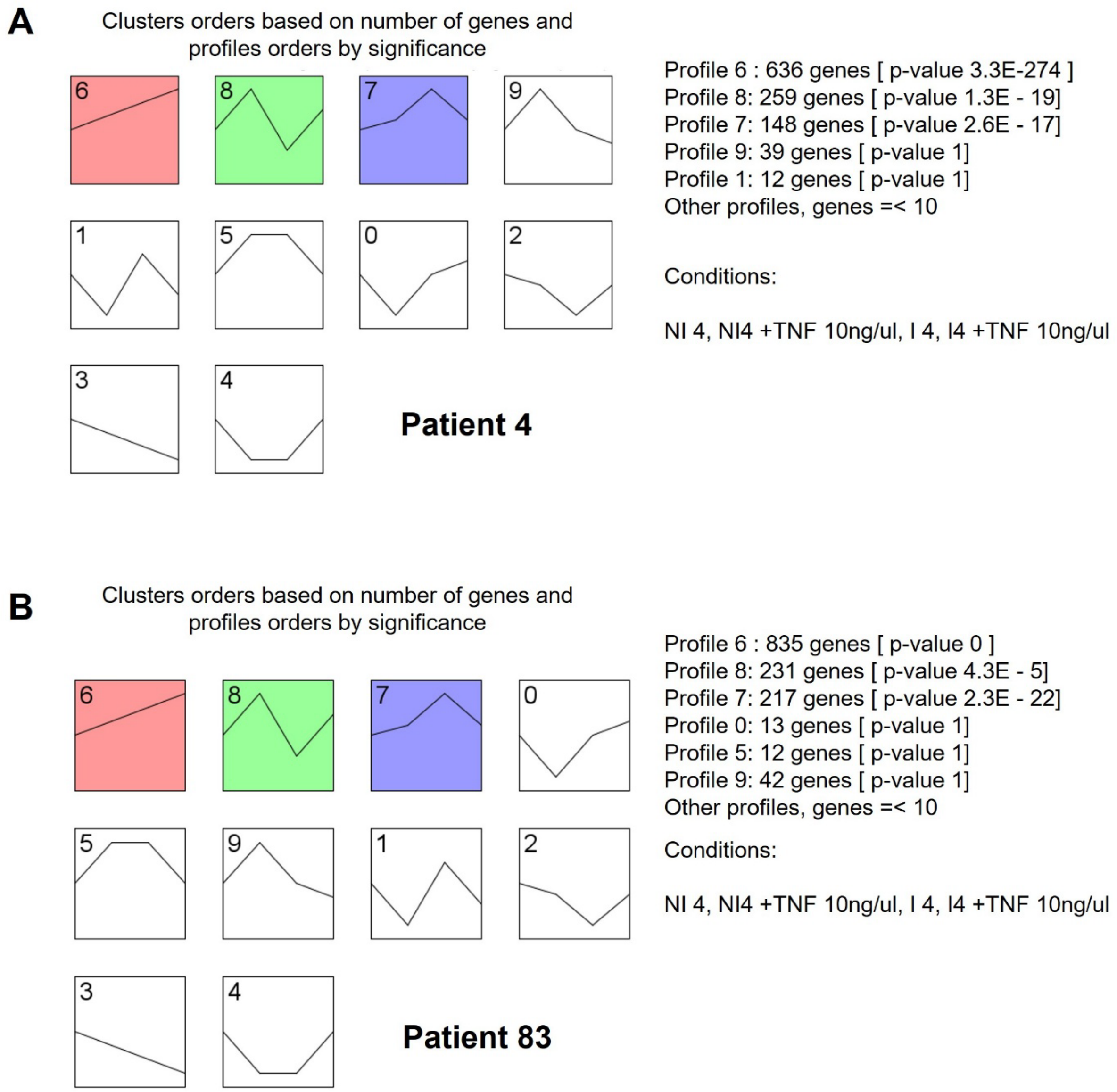

Figure S7. Consistent Inflammation-Specific Transcriptional Reactivation in Two Patients

(**A–B**) STEM clustering of gene expression changes in response to TNFα in NI and I organoids from Patient 4 (**A**) and Patient 83 (**B**). Profile 6 shows consistent upregulation in I organoids. Significant clusters/profiles are shown in color.

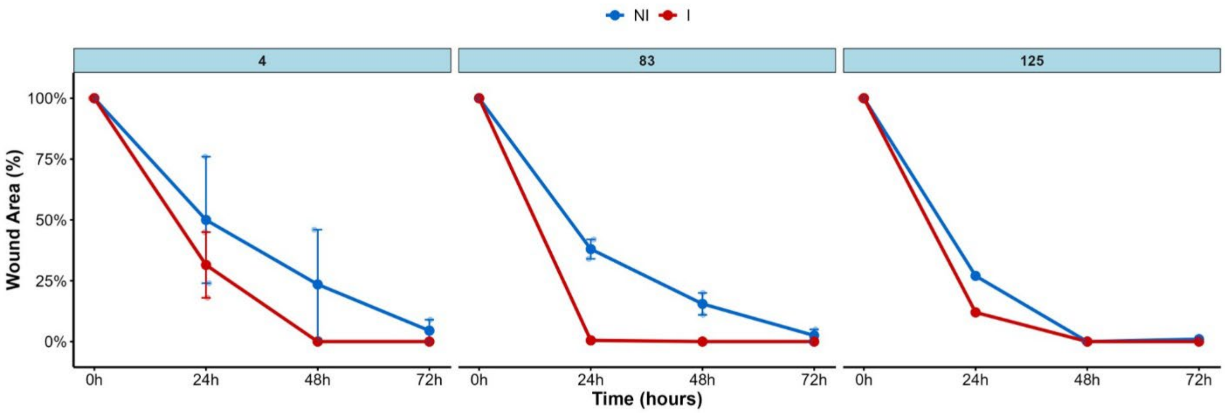

Figure S8. Wound healing across patients. Time-dependent changes in wound area indicate for individual patients show that I colonoid-derived monolayers heal faster than NI colonoid-derived monolayers. Each panel shows one patient with data from two replicates (Pte 125: one replicate).
